# Geometric Specification of Non-Circular Pulleys Made with Various Additive Manufacturing Techniques

**DOI:** 10.3390/ma14071682

**Published:** 2021-03-29

**Authors:** Piotr Krawiec, Dorota Czarnecka-Komorowska, Łukasz Warguła, Szymon Wojciechowski

**Affiliations:** 1 Institute of Machine Design, Poznan University of Technology, 61138 Poznan, Poland; lukasz.wargula@put.poznan.pl; 2 Polymer Processing Division, Institute of Materials Technology, Poznan University of Technology, 61138 Poznan, Poland; dorota.czarnecka-komorowska@put.poznan.pl; 3 Division of Machining, Institute of Mechanical Technology, Poznan University of Technology, 61138 Poznan, Poland; szymon.wojciechowski@put.poznan.pl

**Keywords:** additive manufacturing, geometric part specification, prototyping, non-circular pulleys, surface morphology

## Abstract

The paper presents the procedure of generating geometrical features on the contours of non-circular pulleys through the selection of materials and technological parameters for easy and efficient production of these parts. Based on the models designed in the computer aided design (CAD) system, several prototype non-standard pulleys were made, which were assessed for functional characteristics and correct operation of non-linear gears. The effect of additive technology on the geometric specification of non-circular pulleys was also assessed. The results showed that thanks to the use of additive methods, the need for costly manufacturing of such wheels with subtractive methods was eliminated. Additionally, it is not necessary to design specialized cutting tools or to use conventional or numerically controlled machine tools to manufacture these wheels. The test results showed that in case of selective laser sintering (SLS) the highest accuracy of mapping (0.01 mm) of geometrical features of the surface was obtained. This result is confirmed by the assessment of the morphology of the surface of the teeth of gears made with this technique, characterized by a uniform structure of the working surface of the wheel while maintaining a high tolerance of the outer profile of gear for selective laser sintering at the level of ±0.03 mm. Research has shown that most of the additive methods used to manufacture non-circular pulleys meet the required geometrical features and due to the short production time of these pulleys, these methods also facilitate quick verification of the designed pulley geometry.

## 1. Introduction

In recent years, thanks to the intensive development of the chemical, electromechanical, food, wood, paper and automotive industries, a number of new materials used for drive belts and pulleys have been developed. Currently, many research and industrial centers research on the development of new construction materials, which can be applied for the construction of geometric belts and pulleys. The belts used in tension transmissions are most often made of polymeric materials or their composites [1,2,3]. As a rule, styrene-butadiene rubber (SBR), nitrile-butadiene rubber (NBR), ethylene propylene diene monomer (EPDM) and natural rubber (NR) are used for drive belts, as well as classic thermoplastics, i.e., polyamide (PA), polyvinylidene dichloride (PVDC), acrylonitrile butadiene styrene (ABS), polyurethane (PU), polyethylene (HDPE), polypropylene (PP), polyoxymethylene (POM) [2,4]. However, when higher strength, thermal and chemical resistance and higher flammability are required from the belts, then polymer composites filled with glass fibers (Nylon 6.6 with glass fiber), carbon fiber (carbon fiber) or poly(lactic acid) PLA-carbon are used.

The problems arising during the operation of machines and devices based on cable drives are often errors occurring at the stage of manufacturing and/or assembly of pulleys. For this reason, there is a need for improving or create novelty of the techniques of pulleys production, such as, e.g., additive methods as alternative techniques of producing non-circular pulleys-toothed wheels. The selection of the appropriate manufacturing technique has a significant impact on the geometrical parameters of the wheels as well as the durability and wear of the traction gear [5,6]. As well as in and load transfer technology, transport and control, it’s important to minimize the other errors such as, e.g., roundness error of the pulleys during the manufacturing stage for drive operation.

**Figure 1:**
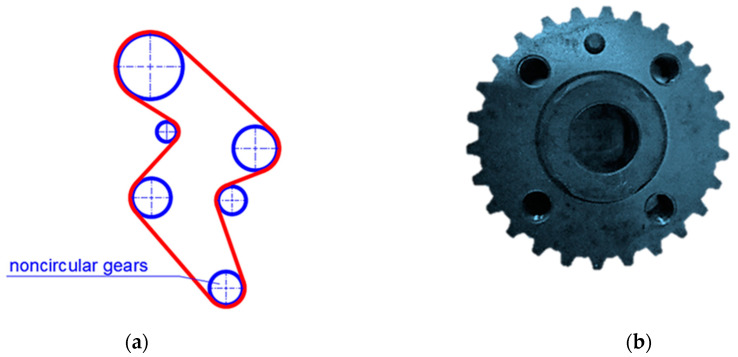
The scheme of a modern internal combustion engine timing system (**a**) with a non-circular wheel (**b**).

This defect causes the so-called phenomenon of a polygon consisting of uneven gear operation. The development of devices for control, feeding and regulation has become the reason for the development of a new design solution of the drive, i.e., gears with a variable ratio or non-rotating gears. In automotive technology, wheels with a non-circular running line, i.e., non-circular wheels (Figure 1) are used. Thanks to the use of an irregularly shaped wheel, optimal conditions for the cooperation between the belt and the wheels in the drive of the engine used, for example, in VW Golf, Audi and other cars are obtained [7,8].

The advantages of the presented solution are the elimination of the toothed belt tensioner (this function is performed by a non-circular wheel) and the improvement of the engine’s smooth running.

Another example of the use of non-round geometry of the non-linear gear actuators can be a bicycle gear drive. Features of this type of drive can be used in the construction of devices described by Branowski [9], Wieczorek [10,11] and Saga [12].

The advantages of using non-parallel traction gears are ensuring the constancy of the designed kinematic features, operational reliability and quiet running. A characteristic feature of these transmissions is the achievement of periodically variable kinematic and dynamic properties thanks to the use of wheels with a non-circular running line. It is very important to efficiently verify the adopted geometrical features of such gears. 

For the production of such atypical wheels, it is possible to use precise methods of material removal treatment [13,14]. However, they were found to be technically feasible but not economically wholly viable [15]. 

In this case, the use of new additive manufacturing techniques for the production of atypical gear teeth may be an appropriate solution. 

Three-dimensional (3D) printing technology has been widely used in various industries. Galeta et al. [16] determined the influence of the structure on the mechanical properties of 3D-printed objects. 

However, there are no known attempts in the literature to assess the geometry and correct functioning of belt drives with non-circular running line pulleys, which were produced by additive techniques. 

One of the elements that determine the choice of manufacturing technique is the shape of the notches made on the rims of the wheels. It depends on whether the gears (transmitting the drive from one shaft to the other) contact with their teeth directly and toothed gears or using a tie in the form of an elastic toothed belt or chain. Like the round pulley machining techniques, the elliptical and oval pulley manufacturing methods are also divided into machining, plastic working, metal powder sintering technology, etc. 

It is very difficult to manufacture wheels with a non-circular running line and what is very important, with variable pitch values around the circumference using the classic methods of wheel production (envelope methods). The variability of the pitches is necessary for the correct cooperation of the toothed belt, which has a constant pitch and the pulley on the circumference, whose variable pitch values must be designed. This problem does not arise with round wheels. On the other hand, if a non-circular pulley with a constant scale value was made, then the belt would not mesh with the pulley. 

The correctness of cooperation between the pulley and the toothed belt must be verified before mass production begins (before making tools, instrumentation, developing the technological process), to avoid negative and costly experiences, such as, for example, making an incorrect shape cutter, chisel or in the case of waterjet cutting to obtain geometric and stereometric features that do not comply with the requirements of the standards. 

Vasko et al. [17] investigated the impact toughness of FRTP composites produced by 3D printing. Blatnicky et al. [18,19] described the use of light metal alloys for the construction of a vehicle frame with an innovative steering mechanism.

Krawiec et al. [20] presented the results of the application of machining of wheels made of aluminum. Methods of assessing the accuracy of the manufacture of toothed pulleys used in non-uniform belt transmissions were applied and the shape of the geometry of the toothed pulley was assessed using a non-contact optical system. 

The cited literature review shows that no attempt has been made so far to produce non-circular pulleys using additive manufacturing techniques. Therefore, it is justified to take up this subject with simultaneous research on the mechanical and geometric properties of the manufactured wheels. Due to the innovative nature of gears with non-circular envelope wheels, it is advisable to produce gears using selected Rapid Manufacturing (RP) methods. The difference between the classic pulleys and those described in this paper results from a different process of belt-pulley coupling. The reason for this is the variability of the scale on the outline of the rolling line, which is related to the impossibility of using classical methods and tools for machining. Thanks to the novel methods proposed in this paper, it is possible to avoid the costly development of atypical cutting tools, the need for specialized machine tools and the excessive time of prototype production. 

The paper presents for the first time in literature, a study concerning an assessment of the geometric features of non-circular wheels produced by various additive technologies, as well as verification of the correctness of the designed CAD model. In the case, of circular pulleys the scale on the wheel tread is constant, but in the case of wheels with a non-circular envelope, the scale on the tread must be variable and dependent on the instantaneous wheel radius. That is why it is so important to verify the designed geometric features with the use of incremental methods.

## 2. Materials and Methods

### 2.1. Design Methods

The frictional engagement process in a gear with non-circular pulleys is significantly different from the rule commonly used in belt transmissions with conventionally shaped pulleys. The dissimilarity of the coupling process results directly from the geometric features of the pulleys (which can be, for example, elliptical, oval, regular, irregular) and their kinematic features. At the stage of designing the geometry of non-circular pulleys in the CAD system, the variation of the pitches on the pulley should be taken into account. The production of a wheel with a variable pitch value by classical methods of gear processing is a difficult process. It is known that the use of, e.g., one shaping tool with the same contour for the entire wheel will fail the contour of the groove or its damage. 

The profiles of the wheels with variable pitch values on the outer contour were plotted in AutoCAD. Then, using the file import functions, their solid models were built in Autodesk Inventor. The 3D models of pulleys were saved in the “stl” format and this form they were exported to the RP machines. 

In the case of using shaping tools, the tooth outline is created without the shaping movement, as a representation of the cutting-edge projection in the workpiece. The use of shaping tools is recommended for large-scale machining. When machining wheels with hobbing tools, the involute contour of the teeth is formed as an envelope of successive tool positions while rolling without slippage of the tool and workpiece rolling lines. Modular cutters are mainly used for making toothing with an intermediate geometric accuracy and large modules that cannot be machined with another tool, on universal. This process is being conducted on special machine tools.

The use of the shape method is particularly recommended in the small-lot production of non-circular gears and pulleys; therefore, the wheels with a non-circular running line were made using Rapid Prototyping methods. 

Due to the symmetry of the elliptical circle, the angle of deviation of the wheel ribs (counted in degrees from the positive values of the vertical axis counterclockwise) was determined only for eleven points. 

**Figure 2:**
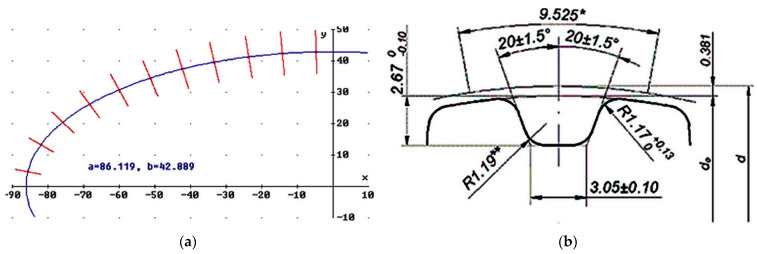
Elliptical wheel: outline of a wheel with the normal positions plotted at the points defining the tooth gaps (mm) (**a**), the outline of a wheel groove for a scale type L (mm) (**b**)**.**

Figure 2a shows the normal positions determined in the Derive program at the points being the centers of the notches and the geometric form of the wheel groove for an L-type pitch is illustrated in Figure 2b. Examples of data needed to develop a tool control program are presented in Table 1.

**Figure 3:**
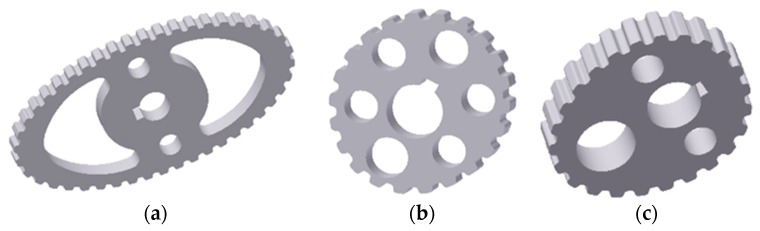
Non-circular wheels of non-rotating gears; (**a**) a wheel with an ellipse-shaped envelope, (**b**) a wheel with an envelope close to a circle, (**c**) a non-circular irregular wheel.

For non-circular irregular wheels, the determination of these values is possible with the use of Bézier or spline curves. The process of modeling the envelope of non-circular wheels with the use of these curves has been described in the works [21,22]. An alternative approach, taking into account the fact that few universal computerized numerical control (CNC) machine tools enable the generation of the code controlling the operation of machines on the basis of C3 class curves, may be to approximate the wheel envelope with a set of strictly tangential wheels. In this paper the three non-circular wheels made with various RP techniques were manufactured (as illustrated in Figure 3).

The first is elliptical in shape, the second is a non-circular regular wheel (it has a symmetry plane) with a slight out of roundness and the third is an irregular non-circular wheel (no symmetry plane). The accuracy of mapping the geometric features of the wheels was adapted on the basis of standards [23,24], relating to round pulleys. On the other hand, the requirements for the stereometric features of the surfaces of the wheels after machining were determined on the basis of the analysis of cooperation between the non-circular pulley and the toothed belt.

### 2.2. Workpiece Materials

Three wheels with a non-circular envelope, produced by various rapid prototyping methods, were selected for the tests, which were compared with a C45E steel wheel with a slightly non-circular envelope, made by machining (Figure 1a) and commonly used in the timing drive of an internal combustion engine. The permissible loads appearing on this kind of non-circular wheels is equal to approx. 400 N in the circumferential direction. The selection of appropriate materials is very important for the target effect of shaping elements using RP techniques. These issues were described by Leturia et al. [25], as well as Rojek et al. [26].

The wheels are made of different materials, depending on the manufacturing technique. In the case of the fused deposition modeling (FDM) technique, a thermoplastic filament was used, i.e., acrylonitrile-butadiene-styrene (ABS) [27]. The ABS is a thermoplastic material characterized by high stiffness and impact resistant, as well as high mechanical stress (tensile strength at 31.46 MPa, Young’s modulus at 1.8 GPa). For the method of the Z Corporation company, the material designated as Zp 130 was used. This is the high-performance composite powder, which is composed of vinyl polymer, sulfate salt and plaster that contains <1% crystalline silica [28]. However, for the selective laser sintering (SLS) technique, a material based on DirectMetal 20 powder was applied [29]. DirectMetal 20 (DM 20) is a grained bronze-based metal powder with a maximum operating temperature of 400 °C. It is a fine-grained mixture of metal powders with a composition close to bronze (Ni-bronze). It consists of the following elements: Cu, P, Sn and Ni. The low content of the low-melting Cu-P blend in combination with bronze forms the binding phase. The resulting parts offer good mechanical properties such as tensile strength up to 700 MPa, Young’s modulus at 80 MPa, hardness 110 HB combined with excellent detail resolution and surface quality (remaining porosity at min. 8%) [30].

### 2.3. Characterization of Manufacturing Methods 

A 3D printer of the Dimension BST 1200 type (Dimension Eden Prairie, MN, USA) was used to make the wheels using the FDM method [31]. The wheels were printed on a 3DP printer (Z Corporation 450, Rock Hill, SC, USA) [32]. The SLS method was tested using an EOSINT M 250 XT 3D printer (EOS GMBH, Krailling, Germany) [33].

**Figure 4:**
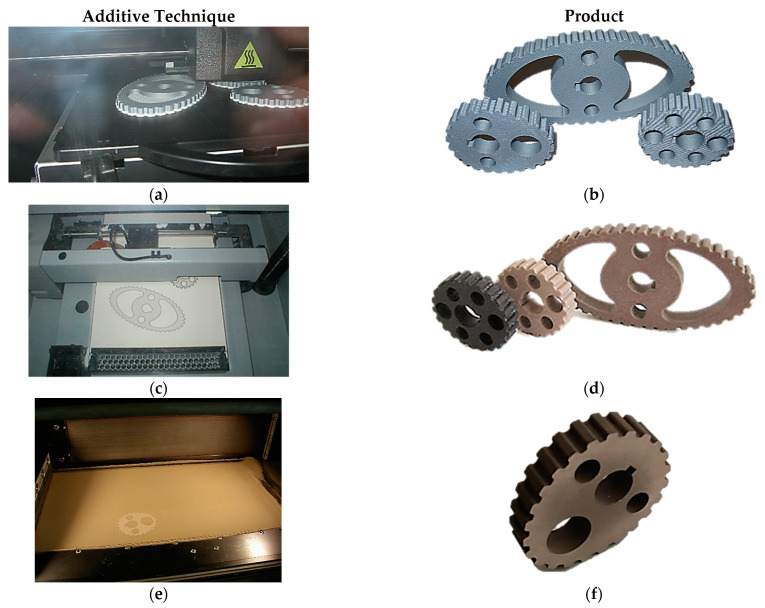
RP techniques employed in the experiment: (**a**) shaping process of non-circular belt pulleys shaped with FDM method, (**b**) Non-circular belt pulleys shaped out with FDM method ABS, (**c**) shaping process of non-circular belt pulleys with 3D printing methods, (**d**) Non-circular belt pulleys shaped with Z Printer 450 method (Zp 130), (**e**) Shaping process of non-circular belt pulleys with SLS method, (**f**) non-circular belt pulleys formed with SLS method after centrifugal blasting (DirectMetal20).

One of the generally used methods in rapid prototyping processes is fused deposition modeling (FDM) which is based on laminar putting of fused model and supported materials with the aid of two nozzles. The generally used material in this process is ABS thermoplastic polymer. In the manufacturing process of non-circular wheels shown in Figure 4a, the Dimension BST 1200 device of Dimension Printing company (Figure 4b) was used. In the case of the FDM method, the horizontal orientation of the element in the working chamber was assumed. Thanks to this, the minimum production time was achieved due to the smallest dimensions of the charge in the Z axis and the lowest possible consumption of support material. In addition, the greater accuracy of the element was obtained. The standard thickness of the deposited layer for this method was assumed to be 254 µm. The interior filling type “solid” and “basic” support were used.

The three-dimensional printing (3DP) method originally developed at the Massachusetts Institute of Technology consists of applying a thin layer of powder to the platform on which the head dispensing the glued material moves. An element is created by overlapping successive layers. After the process is completed, the remaining powder is removed and the model is hardened. Obtaining the desired accuracy of imaging the geometric features and stereometry of the surface of the finished products depends on the appropriate selection of materials. Non-circular pulleys that were made using the Z Printer 450 device by Z Corporation (Figure 4c) are shown in the Figure 4d. In the case of the 3DP method, a layer thickness of 100 μm was assumed and the infiltration depth was 3 mm. 

Another method used in the work was the selective laser sintering (SLS) technique, consisting of local sintering or partial melting of the processed material using a CO_2_ laser beam. The following operating parameters were adopted for the SLS method: laser power 270 W, scanning speed: 3.0 m/s, thickness of the applied powder layers 40 μm, the height of the support structure 2.48 mm, the height of the element with the support structure 17.36 mm. The production parameters were adopted based on the recommendations of the device manufacturers and the experience of the authors.

The application of successive layers takes place in an automatic mode proposed by the program or based on the designer’s experience. The working materials used in selective laser sintering processes can be metals and their alloys in powdered form, e.g., nickel steel, bronze, cobalt-chrome alloys, stainless steel, pure titanium and titanium-aluminum alloys. The wheel shown in Figure 4f is made by Eosint M 250 XT machine and EOS molds (Figure 4e) at the Institute of Advanced Manufacturing Technologies in Krakow.

It should be noted that in the case of the SLS method, only one type of non-circular wheel was produced. However, the thickness and contours of all types of non-circular wheels were the same, therefore, from the point of view of the experiment, it was not necessary to manufacture them all. The workpiece material constituted DirectMetal20 (bronze-based material). 

After completing the modelling process, it is necessary to clean and then sand the surface of the element in contact with the plate on which the forming process was performed. In addition, centrifugal shot blasting was performed for the non-circular pulley. The SLS method can be used to shape products that can be directly used in devices for controlling transport, production and technological processes. It is possible to make combined models that contain complex recess undercuts and internal transitions. Typical applications are main components requiring high mechanical properties-structural components, tools, injection molds, die casting molds. Elements made with the SLS method showed new possibilities for building prototypes for medical applications (orthodontics, implantology, dentistry, etc.) and in the aviation and defense industries.

Selected physical features of the models were assessed, i.e., specific density and Brinell hardness. The density of the models was measured using the hydrostatic method according to PN-EN ISO 1183-1: 2013 [34], using an electronic scale (AXIS AD50-AD200, AXIS. Gdańsk, Poland). The surface hardness of the wheels was measured by the pressed ball method according to PN-EN ISO 2039-1: 2004 [35] using a KB Prüftechnik type KB150R hardness tester (Hochdorf-Assenheim, Germany). The obtained hardness and density of the manufactured wheels are presented in Table 2. The thickness of the wheels was designed to be 15 ± 0.1 mm, which was referenced to a C45E wheel whose thickness was 25 mm. The additively manufactured wheels had lower thickness, which contributed to the lower manufacturing time, but on the other side, it did not contribute to the accuracy of the conducted experiments which have been focused on the evaluation of wheels geometric specification. 

The HB hardness results showed that the hardness of the SLS composite wheels is 374 N/mm^2^, which means that it is the highest compared to the FDM wheel (56 N/mm^2^). It was found that the HB hardness by the pressed-ball method of the printed wheels is much lower compared to the wheel made by machining, which results from the grinding wheel production technology and the type of printing material used. In the case of the FDM wheel made of ABS filament, the model density is 1.04 g/cm^3^, while for the selective laser sintering (SLS) technique, the powder packing density is much higher, about 8 g/cm^3^.

### 2.4. Measurements of Mechanical Properties and Geometric Features of Manufactured Wheels 

The surface morphology of the wheels model was measured using Opta-Tech (Opta-Tech. Warsaw, Poland) microscope. The manufactured pulleys were observed with a camera at a magnification of 20 times. 

The Micro CT equipment type GE Phoenix s240 (Baker Hughes. AGE COMPANY, Houston, TX, USA) has been employed [36]. This device consists of two X-ray tubes. The first high-power directional lamp allows to work with voltages up to 240 kV and obtain an X-ray beam power up to 320 W and a maximum magnification of the measured object up to 100 times. The second lamp, on the other hand, works with much lower power, up to 15 W, but is magnified up to 200 times. The 3D measurements of the models were made with the use of a fast and precise ATOS 3D optical scanner (GOM GmbH Braunschweig, Germany) [37], using the structured blue light technology ensuring precise scanning with high resolution and high speed. Contact measurements of the geometrical features of the wheels were carried out with a Carl Zeiss Contura G27/10/6 coordinate machine (Carl Zeiss AG, Oberkochen, Germany). [38]. The wheel surface roughness measurement was performed using a Form Talysurf Series 50 Rank Taylor Hobson Ltd. stationary profilometer (Taylor Hobson. Leicester, GB) [39]. Five measurements were made for each wheel each time. The parameters of the surface roughness of the wheels on three profiles perpendicular to the axis of wheel rotation were tested. Two software programs from different manufacturers were used in this study: AutoCAD 2010 (Autodesk, San Rafael, CA, USA) and Autodesk Inventor Professional software. 

## 3. Results and Discussions

### 3.1. The Surface Morphology of the Manufactured Parts

**Figure 5:**
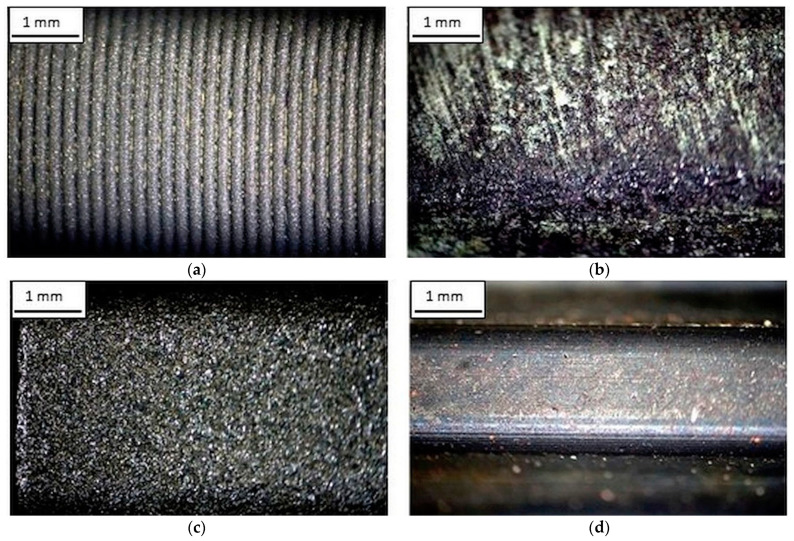
Optical light microscopy images of the wheels surface manufactured with the use of various technologies: (**a**) FDM, (**b**) 3DP Z corporation, (**c**) SLS and (**d**) wheel made of C45E steel with a geometry similar to the circle produced by machining.

The quality of the printed structures depends on various factors such as fiber and powder properties, particle size and shape, density, roughness and porosity [40]. The surface morphology of the manufactured wheels using RP techniques was assessed using the microscopic technique, the results of which are shown in Figure 5.

Figure 5 shows the morphology of the surface of the gear teeth made using the RP (a, b, c) and machining method (d). Figure 5 shows significant differences in the directivity of the roughness on the machined surface and the geometric structure of this wheels. The wheel made by the FDM method (Figure 5a) is characterized by a typical layered structure resulting from the technique of point spreading of the molten thermoplastics polymer, which adversely affects the stereometric features of the wheel. The 3DP printout of the circle (see Figure 5b) shows the even distribution of the composite material layers, which can guarantee favorable geometric features.

In turn, the wheel made by the SLS method (Figure 5c) is characterized by an even and smooth morphology of the working surface of the wheel. This is due to the recrystallization of the higher melting point powder particles, which remain well fixed in volume. As a result, the obtained elements are characterized by good mechanical properties and high manufacturing accuracy and surface quality. 

A machined wheel was used as the reference wheel (Figure 5d). The microscopic observations show that the surface of such a wheel is more even and significantly smoother than the other surfaces of the tested wheels. It results from a precise projection of cutting-edge geometry into the workpiece and selection of relatively low feeds enabling the minimization of formed micro-irregularities onto the machined surface. 

### 3.2. Evaluation of Geometric Specification of Non-Circular Pulleys with the Use of Contact and Non-Contact Methods

**Figure 6:**
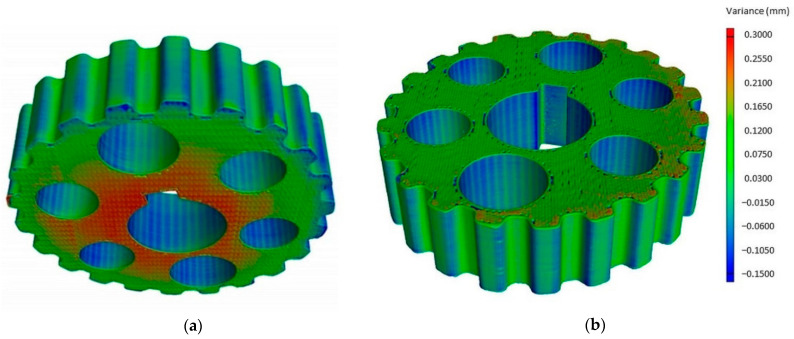
Comparison of the geometric features of the CAD model and the obtained surface model as a result of digitization of a non-circular pulley using a computer tomography: (**a**) upper view of toothed-wheel rim; (**b**) bottom view of toothed-wheel rim.

The gearing of non-circular gears made by various techniques was assessed according to the following criteria, i.e., the correctness of mapping the designed geometric features and surface stereometry, the complexity of preparation of the technological process and the cost of producing a specific batch of wheels. In order to perform these analyzes, procedures for the experimental verification of the correctness of the construction of wheels were developed. Based on the analysis of the literature, it was found that the standardization requirements in terms of the accuracy of mapping geometric features and surface stereometry of non-circular gears and pulleys are unknown. Only the standards for the accuracy of the execution of round pulleys are identified, hence, as part of the study an assessment of geometric features using the non-contact and contact methods was carried out. In the contactless method, two technological solutions were used, the first one used a computer tomography and the second the GOM optical scanner. However, in the contact method, the coordinate Zeiss Contura machine was used and the measurement results were compared to the CAD model of the designed wheels. Figure 6 shows the CAD model of a pulley built in AutoCAD software and Autodesk Inventor (using Autodesk Inventor Professional software) and the surface model obtained by digitizing a non-circular pulley using a computer tomography. 

**Figure 7:**
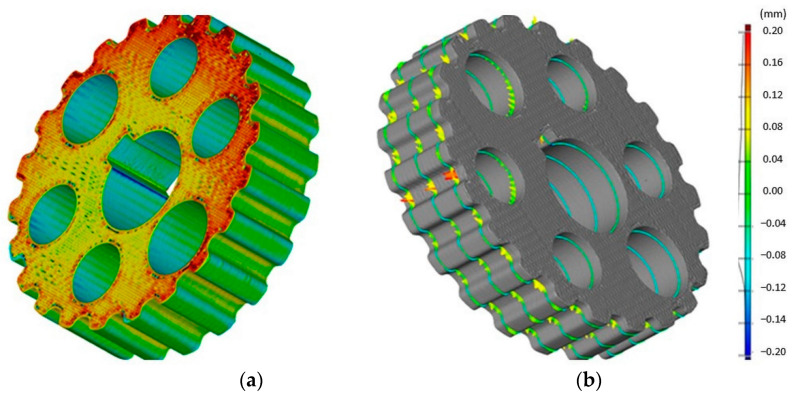
Assessment of the geometrical features of the wheel made with the FDM method using the GOM system (**a**) comparison of the CAD model and the model obtained as a result of scanning, (**b**) the CAD model of the wheel with sections selected to the evaluation.

On the basis of the comparison of these two models and the applied legend, the difference between the CAD model and the surface model obtained as a result of digitization was read. The obtained results for a wheel with a slight non-roundness are shown in Figure 7a. 

The results of the research showed that based on the reference of these values to the recommendations provided in the standards [23,24], correct measurement results were obtained. On the other hand, in Figure 7b the CAD model was compared and the wheel model was made using the FDM method obtained during scanning in the GOM system on the example of selected three cross-sections. It was found that in this case the most important from a practical point of view are the deviations recorded on the tooth surface and the thickness of the wheel is of no significant importance. 

For comparative purposes, it was also decided to perform measurements with the contact method, which enables comprehensive measurements of geometric and stereometric features of machine elements, so that they meet the conditions specified in the PN-84/M85211 and ISO 5294: 2012 standards [23,24]. According to these standards, the tolerance of the parallelism of the teeth in relation to the wheel axis should be 0.001 mm for each millimeter of the wheel rim width, the conicity tolerance of the wheel should not exceed 0.001 mm for each millimeter of the wheel rim width [23,24].

**Figure 8:**
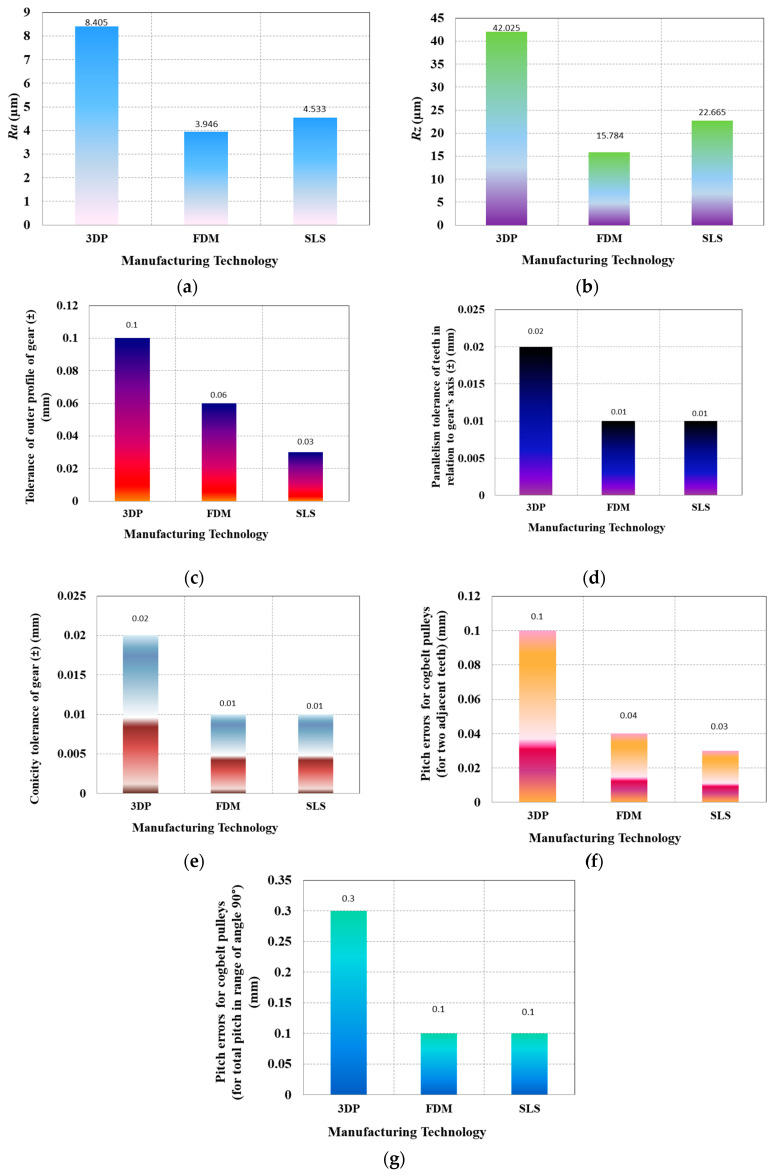
The comparison of various geometrical features for noncircular cogbelt pulleys manufactured with use of additive methods: (**a**) surface roughness *Ra*; (**b**) surface roughness *Rz,* (**c**) tolerance of outer profile of gear; (**d**) parallelism tolerance of teeth in relation to gear’s axis; (**e**) conicity tolerance of gear (for width 0.5”); (**f**) pitch errors for cogbelt pulleys (for two adjacent teeth); (**g**) pitch errors for cogbelt pulleys (for total pitch in range of angle 90°).

The measurement of the external contours of the exemplary pulleys was made in three parallel planes perpendicular to the axis of rotation of the pulley. The process of measuring geometrical features was based on the so-called contour search. The results of measurements of the geometric and stereometric features of the wheels are shown in Figure 8. Based on results presented in Figure 8, one can note that in a majority of investigated geometric part specification and surface roughness indicators, the lowest values of these parameters are found in case of wheels manufactured by SLS technique, followed by ones obtained during manufacturing with FDM and, finally, the values reached in case of wheels produced by 3DP. In case of surface roughness parameters *Ra* and *Rz* the differences are almost 2-fold; however, in case of pitch errors and tolerance of outer profile, differences are even 3-fold. This observation reveals that among the additive techniques, the SLS is characterized by a highest geometrical accuracy towards the manufacturing of non-circular cogbelt pulley.

Surface roughness height values described by an *Rz* parameter, obtained in the case of wheels produced by tested additive manufacturing techniques reach relatively high values up to above 42 μm. In comparison, the surface roughness height levels reached during the machining processes with defined geometry tools (e.g., turning process) are very often lower than 5 μm [14], which can impose a necessity to machine the additively manufactured surfaces by an additional finishing process (e.g., grinding).

The results showed a significant influence of the manufacturing technique on the geometrical features and surface morphology of the pulleys. The applied incremental techniques differ fundamentally in the principle of printing as well as the type and form of the material. It is the type of material that significantly affects the mechanical and thermal properties as well as the structure of the printed pulley. On the other hand, the mechanical properties of products significantly depend on the orientation of the applied layers [41]. It should be noted that the obtained geometric accuracy of non-circular wheels produced by the tested AM methods is significantly dependent on the type of selected workpiece material. This is primarily affected by the material structure and mechanical properties, which influentially affect the level of material shrinkage during the additive process and, thus, product dimensional accuracy.

The FDM technique is a cheap and simple method in which a 3D geometry is created by cutting the model along the XY plane and folding the resulting individual layers along the Z axis, with extruded thermoplastic fibers such as ABS [41]. As a consequence, it gives high accuracy of tooth reproduction. A 3DP printing, as a digital freeforming processing technology provides an opportunity for microstructure controlling and optimizing [42].

**Figure 9:**
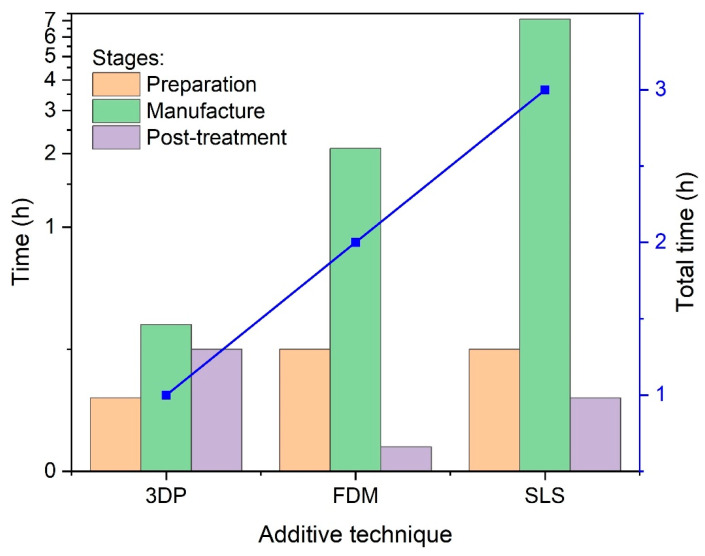
Comparison of the time at stages (preparation-manufacture-post-treatment) and the total time of pulley production using 3DP, FDM and SLS techniques.

Figure 9 compares the total time of a pulley production using various incremental methods, i.e., 3DP, FDM and SLS. The total production time has been measured under real conditions, using the chronometer and consisted of three sub-steps from preparation, through production and finishing. The preparation time was assumed to be the time from the moment the machine operator receives the 3D CAD model to the start of hardening of the first layer of material. Manufacturing time is the time it takes to build a model in individual devices. The post-processing time, on the other hand, is the time between the removal of the element from the machine and its delivery for packaging and shipping. For the 3D technique, it includes the hardening stage, for FDM removal of the substrate and for SLS removal of the plate on which the model was built and shot blasting. 

The analysis of the wheel production times presented in Figure 9 shows that the preparation of elements for production by 3DP and FDM methods are comparable (1.1–2.7 h), while the production time using the SLS method is 8h, which means that it is definitely the longest; however, it is characterized by the highest accuracy of the wheel. 

## 4. Conclusions

The paper analyzes the influence of fabrication techniques on geometric and stereometric features of pulleys and surface quality features, i.e., surface hardness and morphology. The test results showed that additive manufacturing methods, such as 3DP, FDM and SLS, can be successfully used to make prototypes of non-circular toothed pulleys. Based on the research and analysis of the results, the following conclusions were drawn:

- It is not recommended to make prototype non-circular pulleys using the 3D printing method because the accuracy of the tooth profile at the level of ±0.1 mm prevents the correct installation of the toothed belt. This is due to the technology of layering the composite material of the sintered powder.
- The FDM method guarantees a much greater accuracy of the tooth representation (± 0.06 mm), which makes it suitable for the initial assessment of the correctness of geometric features. This is due to the fact that FDM specimens show anisotropic mechanical properties since they vary with filament extrusion direction.
- In the case of the selective laser sintering (SLS) technique, the tooth reproduction accuracy was 0.01 mm of geometric and stereometric surface features, which means the highest surface quality. This result is confirmed by the assessment of the morphology of the surface of the teeth of gears made with this technique, characterized by a uniform structure of the working surface of the wheel, while maintaining a high tolerance of outer profile of gear for SLS at the level of ±0.03 mm.
- The conducted geometric part specification tests revealed that the prototype non-circular pulleys made with the FDM and SLS methods are characterized by the relatively highest geometric quality. As a consequence, they can be employed in a non-parallel belt transmission without the torque load (only frictional resistance) and thus, used in a belt transmission operating under working conditions.

In subsequent scientific works, the authors are planning to verify the designed and manufactured wheels for pulleys at the testing stands.

## Figures and Tables

**Table 1:** Summary of data for building a CAD model of an ellipse wheel.

*x_j_* (mm)	*y_j_* (mm)	*α_j_* (°)
−4.752	42.823	6.3293
−14.235	42.298	18.600
−23.670	41.236	29.862
−33.330	39.540	40.127
−42.261	37.369	48.514
−51.291	34.452	56.110
−60.025	30.754	62.871
−68.292	26.128	69.062
−75.791	20.364	74.960
−81.921	13.226	80.828
−85.610	4.6520	86.889

**Table 2:** Selected physical properties of non-circular wheels.

Symbol	Material	Thickness (mm)	Ball Hardness (N/mm^2^)	Density (g/cm^3^)
FDM	ABS	15 ± 0.1	56.0 ± 4.2	1.040 ± 0.011
3DP	Zp130	15 ± 0.1	114 ± 7.5	1.517 ± 0.014
SLS	DirectMetal20	15 ± 0.1	374 ± 25.0	6.962 ± 0.020
Machining	C45E steel	25 ± 0.1	390 ± 25.1	7.850 ± 0.018

## Data Availability

The data presented in this study are available on request from the corresponding author.
